# Evolution of Sequence Type 4821 Clonal Complex Meningococcal Strains in China from Prequinolone to Quinolone Era, 1972–2013

**DOI:** 10.3201/eid2404.171744

**Published:** 2018-04

**Authors:** Qinglan Guo, Mustapha M. Mustapha, Mingliang Chen, Di Qu, Xi Zhang, Min Chen, Yohei Doi, Minggui Wang, Lee H. Harrison

**Affiliations:** Fudan University Huashan Hospital, Shanghai, China (Q. Guo, M. Wang);; University of Pittsburgh School of Medicine, Pittsburgh, Pennsylvania, USA (M.M. Mustapha, Y. Doi, L.H. Harrison);; Shanghai Municipal Center for Disease Control and Prevention, Shanghai (M. Chen, X. Zhang, M. Chen);; Fudan University Shanghai Medical College, Shanghai (D. Qu)

**Keywords:** Neisseria meningitidis, ST-4821 complex, clonal complex, CC4821, serogroup, genetic recombination, bacterial infections, bacteria, quinolone, China, meningococci, meningitis/encephalitis

## Abstract

The expansion of hypervirulent sequence type 4821 clonal complex (CC4821) lineage *Neisseria meningitidis* bacteria has led to a shift in meningococcal disease epidemiology in China, from serogroup A (MenA) to MenC. Knowledge of the evolution and genetic origin of the emergent MenC strains is limited. In this study, we subjected 76 CC4821 isolates collected across China during 1972–1977 and 2005–2013 to phylogenetic analysis, traditional genotyping, or both. We show that successive recombination events within genes encoding surface antigens and acquisition of quinolone resistance mutations possibly played a role in the emergence of CC4821 as an epidemic clone in China. MenC and MenB CC4821 strains have spread across China and have been detected in several countries in different continents. Capsular switches involving serogroups B and C occurred among epidemic strains, raising concerns regarding possible increases in MenB disease, given that vaccines in use in China do not protect against MenB.

The incidence of meningococcal disease and *Neisseria meningitidis* strain distribution vary over time, within and between countries and regions (*1*). Six serogroups (A, B, C W, X, and Y) account for nearly all cases of invasive meningococcal disease (IMD) globally (*1*). Serogroup C (MenC) cases were rare in China until 2003–2005, when several MenC outbreaks were reported in Anhui Province (*2**–**4*). These outbreaks were caused by a previously unreported hypervirulent clonal complex (CC) 4821 lineage (*5*). A pharyngeal carriage survey and national public health surveillance during 2004–2005 identified CC4821 among sporadic IMD case-patients and asymptomatic carriers across 11 provinces, demonstrating the wide geographic distribution of CC4821 (*2*). During 2005–2012, MenC CC4821 became the leading cause of endemic meningococcal disease in China (*6*). Further analyses of historic isolate collections identified MenB and MenC CC4821 from carriage surveys in the 1970s (*2**,**7*). These studies demonstrated that CC4821 had been mostly associated with asymptomatic carriage over several decades before it emerged as a main cause of IMD (*2**,**7**,**8*). Also, our recent analyses of quinolone resistance among historic meningococcal isolate collections in China found a substantial temporal shift toward increased quinolone nonsusceptibility from the prequinolone era (before ≈1985) to the quinolone era (none versus >70%), particularly within hypervirulent CC4821 and CC5 lineages (*7*). Such findings support the hypothesis that quinolone resistance could have played a role in the emergence of MenC CC4821 outbreaks in China.

Meningococci have a dynamic genome that evolves rapidly through point mutations and frequent recombination. Such genetic changes give rise to strains with novel capsular or other major surface antigens that evade existing population immunity (*9*). A study by Zhu et al. found extensive genomic variation among 22 CC4821 invasive and carriage isolates from 12 provinces in China during 2005–2011 (*8*). In that study, CC4821 isolates belonged to 2 distinct phylogenetic groups, and results indicated that group 1, containing the epidemic reference strain 053442, might be more invasive than group 2 and that MenB and MenC coexisted within both groups 1 and 2 (*8*). 

Our study describes phylogenetic relationships within a collection of historic and current isolates from Shanghai in the prequinolone and quinolone eras and explores how the isolates fit into the larger genetic profile of CC4821 from China (*8*). We aimed to shed light on the genomic factors underlying the abrupt transition of this lineage from a minority strain to a leading cause of endemic disease and outbreaks.

## Materials and Methods

### Strain Selection and Molecular Typing

A total of 374 meningococcal isolates were collected from IMD case-patients, close contacts, and asymptomatic carriers during pharyngeal carriage surveys and routine, laboratory-based public health surveillance conducted during 1965–1985 and 2005–2013 (*7*). The interruption during 1986–2004 was because of the decreased incidence of meningococcal cases and the increased ability to identify *N*. *meningitidis* in hospitals, necessitating fewer isolates to be referred (*7*). In all, 52 CC4821 isolates were identified and underwent molecular characterization using traditional PCR sequencing of the *porA*, *porB*, *fetA*, *fHbp*, *nadA*, *nhba*, and *gyrA* genes and pulsed-field gel electrophoresis as described previously (*7**,**10**,**11*). We selected 8 of these 52 isolates, representing strains from different periods, serogroups, or pulsed-field gel electrophoresis groups, for whole-genome sequencing (WGS) and in-depth phylogenetic analysis. We downloaded assembled contiguous genome sequences (contigs) for 24 additional genome sequences from previous studies of CC4821 in China from GenBank (NM11003, accession no. NZ_ANBU00000000) (*5**,**8*). Therefore, a total of 32 whole-genome sequences underwent core genome phylogenetic analyses for this study (Technical Appendix Table 1).

### Genome Sequencing and Assembly

We performed single molecule real-time sequencing (PacBio; Pacific Biosciences, Menlo Park, CA, USA) on 4 CC4821 isolates (NM040, NM062, NM205, and NM323) and sequenced the remaining 4 isolates (NM001, NM050, NM193, and NM313) by using Illumina HiSeq paired-end sequencing (Illumina, San Diego, CA, USA). We assembled PacBio genomes by using HGAP 4.0 (https://github.com/PacificBiosciences/Bioinformatics-Training/wiki/HGAP) and Illumina genomes by using SPAdes 3.7 (*12*). We annotated assembled contigs by using the Prokka v1.11 (*13*) pipeline and submitted them to the PubMLST *Neisseria* genome database (http://pubmlst.org/neisseria) with ID numbers 41414–41421, where allelic numbers were assigned to all identified genes (*14*).

### Phylogenetic Analyses

We aligned assembled genomes (n = 32) and produced a core genome phylogenetic tree with 1,000 rapid bootstrap replicates by using RAxML and Mauve 2.3 as previously described (*15**–**17*). Serogroup A reference genome Z2491 was included as an outgroup (*18*). We aligned gene sequences selected for focused analyses with MEGA 5.2 (http://www.megasoftware.net) and constructed maximum-likelihood phylogenetic trees under the HKY model of evolution (*19*). We included comparison sequences from a global collection of 133 genomes from the PubMLST *Neisseria* genome database representing all major invasive disease lineages in some of these phylogenetic analyses as references.

### Recombination

We assessed recombination by using ClonalFrameML and Gubbins (*20**,**21*). We then mapped the recombination to the CC4821 reference genome 053442 (online Technical Appendix).

### Gene Content

We assessed gene content by using the Roary 3.6 ortholog clustering program (*22*), which identifies presence or absence of orthologous gene sequences using a cutoff of 90% sequence identity (–i = 90). We defined core genes as genes present in >90% of the genomes. When comparing the gene content of 2 groups of genomes, we defined a gene as specific to that group if it was present in >90% of the genomes in the group and in <20% of the genomes in the comparison group. We downloaded gene functional annotation from the COG database (*23*) and compared genes containing recombinant sequences with nonrecombinant ones based on the major COG classes (cellular processes and signaling, information storage and processing, metabolism, poorly characterized) using uncorrected χ^2^ tests.

## Results

Out of 52 Shanghai isolates, 18 (34%) were from 1972–1977 (3 IMD isolates and 15 asymptomatic carriage isolates), and the remaining 34 (23 IMD isolates, 6 isolates from close contacts, and 5 carriage isolates) were isolated during 2005–2013. Most (56%) Shanghai isolates were serogroup C, 42% were serogroup B, and 1 was nongroupable (Technical Appendix Table 2).

### Phylogenetic Analyses of CC4821 Isolates in China

**Figure 1:**
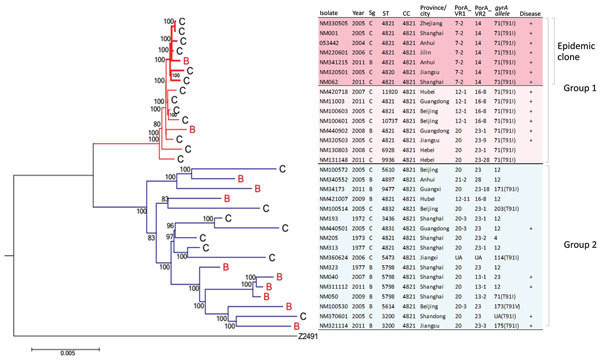
Core genome phylogenetic tree showing relationships between *Neisseria meningitidis* serogroups C and B CC4821 strains, China, 1972–2011. The strains cluster within 1 of 2 distinct phylogenetic groups, group 1 and group 2. Within group 1 is an antigenically distinct clonal group (epidemic clone) containing outbreak-associated strains. Tree is rooted using serogroup A reference strain (Z2491) as an outgroup. Maximum-likelihood phylogenetic trees of aligned core genome sequences were generated under a general time reversible model of evolution with gamma rate heterogeneity, with 1,000 rapid bootstrap replicates represented as a percentage. Only node labels with >80% bootstrap support are shown. Strain type and date and place of isolation are shown; + indicates strains isolated from invasive disease cases. Resistant point mutations on the T91 position of *gyrA* gene are shown alongside data on *gyrA* allele designation. Scale bar represents total substitutions per site. CC, clonal complex; PorA VR, outer membrane protein PorA variable regions; Sg, serogroup; ST, sequence type.

We conducted comparative genome analyses for 8 isolates from Shanghai and 24 publicly available genomes from across China (Figure 1). Among the 8 newly sequenced isolates, 4 were from asymptomatic carriers from 1972–1977 (NM193, NM205, NM313, and NM323), 3 were IMD isolates from 2005–2011 (NM001, NM062, and NM040), and 1 was from an asymptomatic contact of a meningitis patient (NM050). The remaining 24 genome sequences included invasive and carriage CC4821 isolates from 12 provinces across China from 2004–2011, as characterized in previous studies (*4**,**8*). Overall, MenC represented 66% (21/32) of isolates that we analyzed, and the remaining 34% (11/32) belonged to MenB.

The core genome phylogenetic tree classified CC4821 into 2 distinct groups (Figure 1). Group 1 consisted of several isolates that were very closely related; this group contained 15 isolates from 2004 to 2011, and most were from IMD case-patients. In addition, the epidemic reference strain 053442 clustered with a small group of highly similar group 1 isolates. This subgroup is defined as the epidemic clone based on core genome phylogenetic analyses and antigen gene profile (described hereafter). A second more diverse phylogenetic group (group 2) consisted of isolates from Shanghai during 1972–1977 and more recent ones collected from 9 provinces during 2005–2011. In contrast to group 1, only 29% of group 2 isolates were from IMD case-patients (Figure 1). MenB was found in both groups and was interspersed with MenC genomes. Two of 11 MenB isolates belonged to group 1, the remainder to group 2 (Figure 1; Technical Appendix Table 1).

### Characterization of Antigen Gene Content

**Figure 2:**
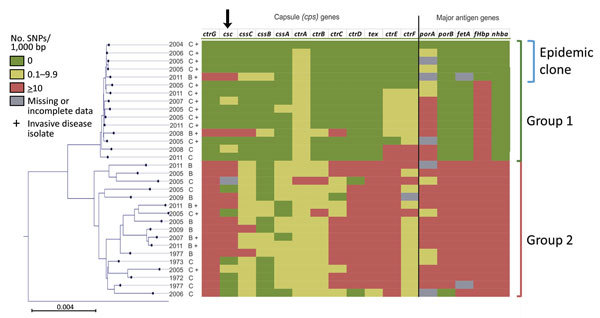
Genetic diversity within capsule and major antigen encoding genes among 32 *Neisseria meningitidis* clonal complex 4821 isolates, China, 1972–2011. Gene sequences from 32 clonal complex 4821 isolates were compared with the epidemic reference strain 053442 (the topmost isolate on the juxtaposed core genome phylogenetic tree). Scale bar represents total substitutions per site. SNP, single-nucleotide polymorphism.

We examined 32 isolates that had undergone WGS to identify the major antigen gene (PorA VR1 and VR2, *porB*, FetA, *nhba*, and *fHbp*) alleles that corresponded to the epidemic strain group 1 or group 2, as determined by the core genome phylogenetic analysis (Figures 1, 2; Technical Appendix Table 1, Figures 1–5). *nadA* was missing in all CC4821 study genomes. Group 1 genomes had diverse *fHbp* and *porA* alleles. All 15 group 1 isolates contained the *porB* 3–48 and FetA F3–3 alleles and *nhba* allele 124. The epidemic clone contained a few highly related *porA* alleles that all encoded unique, conserved *porA* variable regions (PorA VR1 and VR2: P1.7–2, 14). Group 2 isolates had the most antigen gene diversity, containing 5–12 different alleles for each antigen-encoding gene at the nucleotide level and no clear predominance of any single allelic profile. We observed little overlap between the antigen gene allelic profiles in groups 1 and 2. Only 2 of 13 group 1 alleles were also found in group 2. None of the antigen gene alleles found in the epidemic clone was present in group 2, suggesting that the epidemic clone had a nonoverlapping repertoire of antigens compared with historic and current group 2 isolates (Figure 2; Technical Appendix Table 1, Figures 1–5). Group 2 was predominantly associated with PorA P1.20 variants (82%); PorB 3–229 (29%); FetA F3–9 (18%) and FetA F5–2 (18%); FHbp peptide 16 (65%); and *nhba* 553 (41%) (Technical Appendix Table 1).

### Recombination Events Separating Group 1 and Group 2 Isolates

We identified extensive recombination within CC4821 lineage, as detected by ClonalFrameML (*20*). The estimated rate of recombination relative to mutation (R/ϴ) for the entire CC4821 core genome was 1.37 (95% CI 1.40–1.34), and the relative impact of recombination to mutation (r/m) was 20.83, indicating that ≈21 nt were acquired by recombination for every point mutation within the core genome of CC4821. Sublineage specific recombination rates (R/ϴ) were 1.79 (95% CI 1.53–2.05) for group 1, 1.47 (95% CI 1.15–1.79) for group 2, and 0.5 (95% CI 0.26–0.75) for the epidemic clone.

A total of 46 recombination fragments containing 120 genes were mapped to the node that separated group 1 from group 2, indicating that these recombination events could be linked to the divergence of group 1 and 2 sublineages (Technical Appendix Table 3). Sequence alignment and phylogenetic analysis confirmed that the 46 genomic loci represented regions of marked sequence divergence, presumably caused by homologous recombination within a common ancestral genome. These 120 recombinant genes belonged to diverse functional categories; metabolism was the overrepresented functional assignment (51/120 [42.5%]), compared with 28% of nonrecombinant genes (p<0.001 by χ^2^ test). Proportions of other functional groups were similar between recombinant and nonrecombinant genes.

### Recombination Events Unique to the Epidemic Clone

The epidemic clone diverged from group 1 through several additional recombination events, affecting 24 genes across 7 genomic loci (Table). These genomic loci included capsule translocation genes (*ctrE* and *ctrF*); major outer membrane protein (OMP) antigen genes *porA*, *porB*, and *fHbp*; *pts* genes involved in carbohydrate transport and metabolism; and the *tkl* locus encoding DNA polymerase and several metabolic enzymes.

**Table:** Recombinant genes unique to the *Neisseria meningitidis* clonal complex 4821 epidemic clone, China, 1972–1977 and 2005–2013*

Gene ID	Gene name	Annotation
NMCC_0090	*ctrE*	Polysialic acid capsule modification protein LipA
NMCC_0091	*ctrF*	Polysialic acid capsule modification protein LipB
NMCC_0136		Putative RmuC-like protein
NMCC_0137		Putative metallo-dependent hydrolase
NMCC_0138		Putative periplasmic DNA ligase (polydeoxyribonucleotide synthase [ATP])
NMCC_0140	*ptsIIA*	Phosphotransferase system, enzyme IIA (protein IIA)
NMCC_0141	*ptsH*	Phosphocarrier protein HPr (phosphotransferase system, histidine-containing protein)
NMCC_0142	*ptsI*	Phosphoenolpyruvate-protein phosphotransferase (phosphotransferase system, enzyme I; protein I)
NMCC_0158	*porB*	Major outer-membrane protein P.IB (protein IB; PIB; porin)
NMCC_0350		Putative peptidase
NMCC_0351	*fHbp*	Factor H binding lipoprotein (lipoprotein GNA1870)
NMCC_0352	*fba*	Fructose-bisphosphate aldolase
NMCC_1338	*porA*	Major outer-membrane protein P.IA (protein IA; PIA; porin)
NMCC_1341	*greA*	Transcription elongation factor GreA (transcript cleavage factor GreA)
NMCC_1342	*aroA*	3-phosphoshikimate 1-carboxyvinyltransferase (5-enolpyruvylshikimate-3-phosphate synthase; EPSP synthase; EPSPS)
NMCC_1343		Conserved hypothetical lipoprotein
NMCC_1363		Putative DnaQ-like exonuclease
NMCC_1364		Arg tRNA
NMCC_1365		Glu tRNA
NMCC_1366		Putative dioxygenase
NMCC_1367		Conserved hypothetical membrane protein
NMCC_1368		Putative ferredoxin
NMCC_1370	*tkt*	Transketolase
NMCC_2038	*fmt*	Methionyl-tRNA formyltransferase

*Gene IDs correspond to gene identifiers in the clonal complex 4821 reference genome 053442. ATP, adenosine triphosphate; EPSP, 5-enolpyruvylshikimate-3-phosphate; EPSPS, EPSP synthase; ID, identifier.

### Gene Content

We identified a total of 3,292 unique genes, of which 1,730 core genes were shared by most CC4821 genomes. Eleven genes were present in >90% of group 1 isolates but missing in most group 2 isolates. Genes that were predominantly found in group 1 included *lbpB* encoding lactoferrin binding protein B, *nhaP* encoding Na^+^/K^+^ antiporter, and genes encoding several putative enzymes and 4 hypothetical proteins whose functions are unknown (Technical Appendix Table 4). These genes were spread across several locations on the reference genome 053442, suggesting that they were acquired separately rather than in a single event. The epidemic clone had no noticeable gene gain or loss compared with other group 1 isolates.

### Capsular Gene *cps* Cluster

The *cps* cluster of *N. meningitidis* consists of 6 regions (D-A-C-E-D′-B) required for capsule biosynthesis (region A), transport (region C), and translocation (region B) (*24*). The group 1 isolates were characterized by a novel capsule region A (*ctrG4*-*cssE1*-*csc1*-*cssC3*-*cssB1*-*cssA3*), associated with region C (*ctrA5*-*ctrB1*-*ctrC4*-*ctrD1*) and region E (*tex*-*orf1*-*orf2*) Technical Appendix Table 1). Nine of 15 regions A-C-E identified among group 1 isolates were identical, and another 4 were almost identical (1-bp variation in *csc*, *cssA*, or *ctrA* over 14,489 bp). Only 2 diverged because of the allelic replacement containing the serogroup-specific polysialyltransferase gene (*csb*), resulting in capsular switches involving serogroups B and C (NM341215 and NM440902) (Technical Appendix Table 2). Group 2 had substantial genetic diversity within the *cps* cluster, with no 2 isolates possessing an identical *cps* gene allelic profile (Figure 2).

### Characterization of CC4821 Isolates from Shanghai

Our results classified 52 CC4821 isolates from Shanghai into epidemic clone or groups 1 and 2 on the basis of associated *porA*, *porB*, *fetA*, *fHbp*, and *nhba* antigen genes defined by WGS (Figure 1; online Technical Appendix Table 2). A substantial proportion of strains in Shanghai (23/52 [44%]) belonged to group 1, with 0–1 antigen gene differences (Figure 1; Technical Appendix Table 2, Figures 1–5). Shanghai group 1 isolates were exclusively isolated during 2005–2013; were MenC (19/23), MenB (3/23), or nongroupable (1/23); and were isolated from IMD case-patients (18/23), close contacts of IMD case-patients (4/23), or an asymptomatic carrier (1/23). Within group 1, a total of 16 isolates (16/23 [70%]) contained PorA P1.7–2,14 and were consistent with the epidemic clone by all 5 antigen genes. Twenty-nine (29/52 [56%]) Shanghai isolates belonged to group 2 and differed from group 1 by 4–5 out of 5 antigen loci. Shanghai group 2 was dominated by carriage isolates (21/29 [72%]) from 1972–1977 and 2005–2013. MenB equaled or outnumbered MenC among historic (9/18 [50%]) and recent (10/11 [91%]) group 2 CC4821 isolates. MenB strains in group 1 possessed *fHbp* 498 or 22 encoding peptides 1.80 or 2.22 (subvariants belonging to FHbp variant group 1 or 2), as most (17/19 [89%]) of MenC epidemic clones did (Technical Appendix Tables 1, 2).

All group 1 isolates contained fluoroquinolone-resistant *gyrA* allele 71 (corresponding to allele R1 in our previous study [*7*]), which was generated by a nonsynonymous mutation of fluoroquinolone-susceptible *gyrA* allele 12 (allele S1), creating an amino acid substitution of T91I. The *gyrA* allele 12/S1 was carried by 76% (22/29) of the group 2 strains (Technical Appendix Table 2). In contrast, only 4 of 29 group 2 isolates from Shanghai and another 6 publicly available group 2 genomes contained resistant *gyrA* alleles, which were different from each other. Group 2 isolates with genotypic resistance to quinolones were genetically highly diverse as evidenced by diverse sequence types, different antigen gene profiles, and expression of both MenB and MenC capsules. However, all quinolone-resistant isolates were from 2005–2013 (Figure 1; Technical Appendix 1, 2).

## Discussion

This study presents detailed genomic analyses of the current major endemic meningococcal disease lineages in China, serogroups C and B CC4821. Phylogenetic analyses have suggested that the epidemic clone (corresponding to the epidemic China^CC4821-R1-C/B^ clone described in our previous work [*7*)]) responsible for most recent CC4821 disease cases was nested within a distinct phylogenetic group (group 1), consistent with recent clonal expansion of a genetically distinct strain. In contrast, group 2 was temporally and genetically more diverse and accounted for a smaller proportion of IMD cases, as evidenced by the preponderance of asymptomatic carriage isolates within this group (*8*). Group 1 isolates were first identified during MenC outbreaks during 2003–2005, whereas group 2 isolates were mostly associated with carriage from the 1970s to 2013 (*7*).

This work adds to the description of CC4821 by a previous study (*8*) by demonstrating that, within group 1, a genetically distinct clone exists that shares the *porA* antigenic formula P1.7–2,14 and comprises strains associated with outbreaks and a large proportion of epidemic disease cases. This finding is in keeping with surveillance studies reporting MenC strains during 2005–2012 indicating that 55% of 238 confirmed meningococcal disease cases and 84% of 131 MenC strains belong to CC4821 with *porA* P1.7–2,14 (*6*), suggesting that the expansion of CC4821 was caused by clonal expansion of this antigenic type. Genomic analyses demonstrated that the epidemic CC4821 lineage had undergone 2 crucial genetic events compared with historic asymptomatic carriage isolates. First, CC4821 diverged into 2 major sublineages through extensive recombination events predominantly affecting genes involved in metabolic functions. Such extensive allelic exchanges might have enhanced the transmission fitness or the invasive potential within group 1. Second, a virulent, antigenically unique, epidemic strain emerged from within group 1 in a second set of more focused recombination events affecting major antigen genes, *porA, porB, fHbp*, capsule genes *ctrE* and *ctrF*, and several metabolic genes associated with oxidative phosphorylation and glycolytic processes. These genetic changes possibly account for the rapid dissemination and increased invasive potential of the epidemic clone.

This work also adds to the evidence that the emergence and persistence of virulent meningococcal strains occurs through the introduction of a novel antigenic variant in an immunologically naive population (*9**,**15**,**25**–**27*). In addition, even though a few major antigen gene repertoires mediate the microevolution of virulent meningococcal strains, a larger and more assorted number of metabolic genes might be involved in the divergence of sublineages. Additional research is needed to elucidate the intricate interactions between various metabolic pathways in the fitness and virulence potential of meningococci.

Selection pressure of fluoroquinolones might also affect the evolution and adaption of meningococcal strains, as evidenced by various resistant *gyrA* alleles recovered in many meningococcal lineages and singletons only in the quinolone era in China (*7**,**28*). All of the group 1 CC4821 strains contain *gyrA* allele 71/R1, in contrast to various resistant *gyrA* alleles in group 2, suggesting acquisition of this trait by group 1 at an early stage in evolution. The *gyrA* allele 71/R1 derived from *gyrA* allele 12/S1, which was possessed by most of the group 2 strains both in prequinolone and quinolone eras, indicating their common origin. The precise role of quinolone resistance in the emergence of the epidemic clone requires further study.

The relative rates and impact of recombination within the CC4821 lineage were considerably higher than previous estimates that examined recombination across 7 housekeeping genes (*29**–**31*). We identified 4,026 recombination events and 21 recombinant single-nucleotide polymorphisms (SNPs) for every point mutation within CC4821. Estimates of meningococcal recombination rates from whole-genome sequence data are limited. A study of MenA CC5 strains in Africa (*27*), an epidemic lineage notable for relatively low genetic diversity, found 34 recombinant sequences and 12 recombinant SNPs for every point mutation. A study of recombination rates within conserved multilocus sequence type loci among carriage isolates from the Czech Republic in 1993 found 6.2–16.8 SNPs caused by recombination for each point mutation (*29*). These data suggest that although homologous recombination is a shared mechanism for meningococcal strain emergence and persistence (*29**,**31**–**33*), the frequency and extent of recombination likely differs substantially between lineages.

High rates of recombination have led to multiple distinct capsular switch strains expressing group B and C capsule, as described in this study, and serogroup W (*8**,**34*). Both MenB and MenC were phylogenetically diverse and interspersed within both groups 1 and 2, suggesting multiple distinct capsular switches between these serogroups rather than clonal expansion of a single capsular switch strain (*35**,**36*). Moreover, recombination events that led to no apparent change in capsular phenotype were also prevalent.

Wide geographic and temporal spread of MenB strains in China is of concern given that vaccines currently in use do not protect against MenB disease. Furthermore, marked heterogeneity exists among both MenB and MenC strains within the gene encoding FHbp, a key component of MenB vaccines.

At the time of its emergence, CC4821 was confined to China and Taiwan (*37*). However, review of the PubMLST *Neisseria* genome database suggests a recent increase in the geographic spread of CC4821. CC4821 isolates were reported from a small number of asymptomatic carriers in Brazil (2014) (*38**,**39*) and Australia (2012–2017), as well as isolated disease cases and carriers from France (2009–2011), the United Kingdom (2011–2014), the United States (2007–2016), and India (2017) (*14*). A recent case report of a quinolone-resistant sequence type 4821 strain from Japan in a patient with no history of foreign travel also suggests local transmission of CC4821 in Japan (*40*). Global CC4821 carriage and IMD isolates on the PubMLST *Neisseria* database were antigenically diverse, containing PorA types associated with both group 1 and 2 strains, and expressed both serogroup C and B capsules. This finding suggests low-level dissemination of CC4821 strains with diverse virulence and antigenic types as opposed to clonal spread of a single epidemic strain. This pattern is in contrast to the pandemic spread of highly clonal serogroup A epidemic strains from China from the 1960s through the 1990s (*25*). Genome-based surveillance of these global CC4821 strains is needed to monitor the global spread of this clonal lineage.

Our study is limited by lack of isolates before the 1970s and during 1986–2004; isolates from those periods might have provided a clearer picture of the multiple evolutionary steps that led to the epidemic CC4821 clone. Also, the high frequency of recombination within capsular genes makes it difficult to accurately determine the direction of capsular switch.

In summary, we have presented detailed genomic analysis of a major hypervirulent meningococcal lineage associated with MenC and MenB in China and identified key genomic factors that might have led to the emergence and persistence of MenC in China. The potential emergence of MenB is of public health concern. Strengthened laboratory surveillance for disease cases and carefully planned carriage surveys are needed to monitor global trends, detect outbreaks, and inform immunization policies.

Technical Appendix Method for recombination assessment, epidemiologic and genetic characteristics of *Neisseria meningitidis* clonal complex 4821 isolates in China, and genes underlying divergence.
