# Supplementary material for: Evolution of Sequence Type 4821 Clonal Complex Meningococcal Strains in China from Prequinolone to Quinolone Era, 1972–2013
Source: Emerg Infect Dis. 2018 Apr;24(4):683–90. doi: 10.3201/eid2404.171744 (PMC5875256; doi:10.3201/eid2404.171744)
Supplement: Technical Appendix — Method for recombination assessment, epidemiologic and genetic characteristics of Neisseria meningitidis clonal complex 4821 isolates in China, and genes underlying divergence. [file 17-1744-Techapp-s1.pdf]

Article DOI: https://doi.org/10.3201/eid2404.171744

Evolution of Sequence Type 4821 Clonal Complex Meningococcal Strains in China from Prequinolone to Quinolone Era, 1972–2013

Technical Appendix

Recombination

Recombination was assessed using maximum-likelihood inference under the ClonalFrame model of evolution implemented by ClonalFrameML (*1*). Aligned core genome sequences and a RAxML maximum-likelihood phylogenetic tree were used as input for ClonalFrameML under the standard model with 100 simulations (-emsim 100). Additional assessment of recombination was done using Gubbins (*2*) and compared to ClonalFrameML findings for consistency. The relative impact of recombination to mutation (r/m) was calculated from ClonalFrameML output using the formula $r/m = (R/\theta) \times \delta \times \nu$ where R/θ is the ratio of recombination to mutation rates, δ is the mean length of imports and ν, the average distance of the imports as described (*1*).

The output from ClonalFrameML provided detailed breakdown of inferred recombinant sequences for every node of the core genome maximum-likelihood phylogenetic tree. Selected nodes, representing the divergence of group 1 from group 2 CC4821 lineages; and divergence of epidemic strains with P1.7–2 antigenic formula from within group 1 were selected for more in-depth analyses. Inferred recombinant sequences were mapped on to the reference genome 053442, using Blastn and custom Unix shell scripts to identify corresponding gene annotations.

Recombinations within these segments were further demonstrated using sequence alignments and focused phylogenetic analyses using corresponding sequences from several global reference strains for comparison.

References

1. Didelot X, Wilson DJ. ClonalFrameML: efficient inference of recombination in whole bacterial genomes. PLOS Comput Biol. 2015;11:e1004041. PubMed http://dx.doi.org/10.1371/journal.pcbi.1004041

2. Croucher NJ, Page AJ, Connor TR, Delaney AJ, Keane JA, Bentley SD, et al. Rapid phylogenetic analysis of large samples of recombinant bacterial whole genome sequences using Gubbins. Nucleic Acids Res. 2015;43:e15. PubMed http://dx.doi.org/10.1093/nar/gku1196

Technical Appendix Table 1. Epidemiologic and genetic characteristics of 32 *N. meningitidis* isolates belonging to clonal complex 4821 lineage in China[abc]

PubMLST ID	Isolate name	Year	Sg	PorA_VR1	PorA_VR2	*'porB*	FetA_VR	*porA* (NEIS1364)	*porB* (NEIS2020)	*fetA* (NEIS1963)	*nhba* (NEIS2109)	*'fHbp*	FHbp variant[f]	*gyrA* allele
57871	NM330505	2005	C	7–2	14	3–48	F3–3	ND	29	64	124	498	1.80/B	71[d]
58133	NM001	2005	C	7–2	14	3–48	F3–3	56	29	64	124	498	1.80/B	71[d]
12672	053442	2004	C	7–2	14	3–48	F3–3	7	29	64	124	498	1.80/B	71[d]
57851	NM220601	2006	C	7–2	14	3–48	F3–3	1029	29	64	124	498	1.80/B	71[d]
57872	NM341215	2011	B	7–2	14	3–48	F3–3	ND	29	ND	124	498	1.80/B	71[d]
57869	NM320501	2005	C	7–2	14	3–48	F3–3	1030	29	64	124	323	1.276/B153	71[d]
58134	NM062	2011	C	7–2	14	3–48	F3–3	56	29	64	124	22	2.22/A10	71[d]
57862	NM420718	2007	C	12–1	16–8	3–48	F3–3	1031	29	64	124	489	1.419/B	71[d]
41444	NM11003	2011	C	12–1	16–8	3–48	F3–3	1031	29	64	124	489	1.419/B	71[d]
57853	NM100603	2005	C	12–1	16–8	3–48	F3–3	1031	29	64	124	489	1.419/B	71[d]
57863	NM100601	2005	C	12–1	16–8	3–48	F3–3	1031	29	64	124	489	1.419/B	71[d]
57852	NM440902	2008	B	20	23–1	3–48	F3–3	1026	29	64	124	22	2.22/A10	71[d]
57870	NM320503	2005	C	20	23–9	3–48	F3–3	ND	29	64	124	489	1.419/B	71[d]
57855	NM130803	2008	C	20	23–1	3–48	F3–3	1026	29	64	124	22	2.22/A10	71[d]
57854	NM131148	2011	C	20	23–28	3–48	F3–3	1032	29	64	124	22	2.22/A10	71[d]
57858	NM100572	2005	C	20	23	UA	F2–9	640	UA	2612	74	16	2.16/A19	12
57856	NM340552	2005	B	21–2	28	3–460	F2–9	UA	1275	2612	74	39	2.18/A	12
57857	NM34173	2011	B	20	23–18	3–81	F1–33	ND	43	2610	566	21	2.21/A07	171[d]
57861	NM421007	2009	B	12–11	16–8	3–84	F5–7	1035	150	2191	553	16	2.16/A19	12
57860	NM100514	2005	C	20	23–1	3–458	UA	1034	UA	2620	UA	UA	2.16/A19	203[d]
58135	NM193	1972	C	20–3	23–1	3–607	F1–5	1025	1273	2615	966	474	2.404/A	12
57859	NM440501	2005	C	20–3	23	UA	F5–70	1033	UA	2617	966	490	1.420/B	12
58136	NM205	1973	C	20	23–2	3–608	F5–135	643	1274	2616	553	16	2.16/A19	4
58137	NM313	1977	C	20	23–1	3–35	F14–1	1026	44	ND	553	16	2.16/A19	12
57864	NM360624	2006	C	UA	UA	3–48	F4–2	ND	29	255	UA	16	2.16/A19	114[d]
58132	NM323	1977	B	20	23	3–229	F3–9	640	265	1465	553	16	2.16/A19	12
58130	NM040	2007	B	20	13–1	3–370	F5–2	1027	1270	2614	553	16	2.16/A19	23
57865	NM311112	2011	B	20	13–1	3–229	F5–2	1036	265	2614	553	16	2.16/A19	12
58131	NM050	2009	B	20	13–2	3–229	F5–2	1028	265	2614	553	16	2.16/A19	71[d]
57866	NM100530	2005	B	20–3	23	3–229	F3–9	1033	265	1465	1346	16	2.16/A19	173[e]
57868	NM370601	2005	C	20	23	UA	F3–9	640	UA	1465	UA	637	1.547/B	UA[d]
57867	NM321114	2011	B	20	23–3	3–229	F1–124	1037	265	2619	1368	677	1.547/B	175[d]

[a] *nadA* is missing in all of the 32 strains.
[b] Light pink, lavender blush and light cyan indicated strains belonged to the epidemic clone, non-epidemic clones in phylogenetic group 1 and group 2, respectively.
[c] Abbreviations: ND, not detected or incomplete sequence; Sg, serogroup; UA, unassigned; X, missing sequence data.
[d] Amino acid mutation at position 91, T91I.
[e] Amino acid mutation at position 91, T91V.

PubMLST ID	Isolate name	Year	Sg	PorA_VR1	PorA_VR2	'porB	FetA_VR	*porA* (NEIS1364)	*porB* (NEIS2020)	*fetA* (NEIS1963)	*nhba* (NEIS2109)	'*fHbp*	FHbp variant[f]	*gyrA* allele

[f] FHbp variant: Novartis subvariant/Pfizer subfamily or subvariant. There are three major variants (variant 1, 2 and 3) in Novartis classification for FHbp proteins, named with a major variant number and a subvariant number (e.g., 1.80). Pfizer classifies FHbp into two subfamilies, A and B, with assigned number indicating subvariant (e.g., A10).

Technical Appendix Table 1 (continued)

PubMLST ID	Isolate name	Sg	*ctrG*	*cssE*	*csc*	*csb*[c]	*cssC*	*cssB*	*cssA*	*ctrA*	*ctrB*	*ctrC*	*ctrD*	*tex*(NEIS0059)	*orf1*(NEIS2910)	*orf2*(NEIS2854)	*ctrE*	*ctrF*
57871	NM330505	C	4	1	1	X	3	1	3	5	1	4	1	4	1	1	3	3
58133	NM001	C	4	1	1	X	3	1	3	5	1	4	1	4	1	1	3	3
12672	053442	C	4	1	1	X	3	1	3	1	1	4	1	4	1	1	3	3
57851	NM220601	C	4	1	1	X	3	1	3	5	1	4	1	4	1	1	3	3
57872	NM341215	B	13	X	X	325	2	13	3	263	1	4	1	4	1	1	3	3
57869	NM320501	C	4	1	1	X	3	1	3	5	1	4	1	4	1	1	3	3
58134	NM062	C	4	1	1	X	3	1	3	5	1	4	1	4	1	1	770	16
57862	NM420718	C	4	1	UA	X	3	1	3	5	1	4	1	4	1	1	770	16
41444	NM11003	C	4	1	1	X	3	1	3	5	1	4	1	4	1	1	770	16
57853	NM100603	C	4	1	1	X	3	1	3	5	1	4	1	4	1	1	770	16
57863	NM100601	C	4	1	1	X	3	1	613	5	1	4	1	4	1	1	770	16
57852	NM440902	B	5	X	X	1	205	3	3	271	18	7	1	4	1	1	770	16
57870	NM320503	C	4	1	1	X	3	1	3	5	1	4	1	4	1	1	770	16
57855	NM130803	C	4	1	9	X	3	1	3	5	1	4	1	4	1	1	898	533
57854	NM131148	C	4	1	1	X	3	1	3	5	1	4	1	4	1	1	827	532
57858	NM100572	C	12	1	UA	X	2	1	615	272	208	69	19	1087	1	1	151	535
57856	NM340552	B	5	X	X	1	2	1	148	5	1	32	225	1086	1	1	338	16
57857	NM34173	B	229	X	X	UA	2	1	614	5	1	4	1	1086	1	1	828	534
57861	NM421007	B	13	X	X	276	2	1	2	5	2	4	170	1090	1	UA	829	ND
57860	NM100514	C	173	1	1	X	26	1	616	10	209	3	1	1089	1	1	338	506
58135	NM193	C	173	1	1	X	2	1	612	5	2	7	18	1085	1	1	338	16
57859	NM440501	C	173	1	UA	X	2	1	12	5	39	13	45	1088	1	1	338	16
58136	NM205	C	173	1	1	X	203	1	95	5	2	7	18	62	1	1	338	16
58137	NM313	C	173	1	1	X	2	1	2	5	2	7	18	62	1	1	826	16
57864	NM360624	C	173	1	1	X	2	1	3	263	2	12	1	1091	87	1	830	536
58132	NM323	B	237	X	X	308	204	13	2	5	2	7	18	62	1	1	338	16
58130	NM040	B	5	X	X	108	2	13	3	5	2	7	18	62	1	1	338	16
57865	NM311112	B	5	X	X	320	2	13	2	5	2	7	18	62	1	1	338	16
58131	NM050	B	5	X	X	1	285	1	12	5	2	111	18	62	1	1	338	16
57866	NM100530	B	5	X	X	1	2	1	313	273	18	7	226	1092	1	1	338	16
57868	NM370601	C	173	11	1	X	2	13	3	2	210	3	7	1094	1	1	1175	537

PubMLST ID	Isolate name	Sg	*ctrG*	*cssE*	*csc*	*csb*[c]	*cssC*	*cssB*	*cssA*	*ctrA*	*ctrB*	*ctrC*	*ctrD*	*tex*(NEIS0059)	*orf1*(NEIS2910)	*orf2*(NEIS2854)	*ctrE*	*ctrF*
57867	NM321114	B	5	X	X	108	2	13	3	136	2	12	108	1093	1	1	338	16

[a] *nadA* is missing in all of the 32 strains.

[b] Light pink, lavender blush and light cyan indicated strains belonged to the epidemic clone, non-epidemic clones in phylogenetic group 1 and group 2, respectively.

[c] Abbreviations: ND, not detected or incomplete sequence; Sg, serogroup; UA, unassigned; X, missing sequence data.

[d] Amino acid mutation at position 91, T91I.

[e] Amino acid mutation at position 91, T91V.

[f] FHbp variant: Novartis subvariant/Pfizer subfamily or subvariant. There are three major variants (variant 1, 2 and 3) in Novartis classification for FHbp proteins, named with a major variant number and a subvariant number (e.g., 1.80). Pfizer classifies FHbp into two subfamilies, A and B, with assigned number indicating subvariant (e.g., A10).

Technical Appendix Table 2. Epidemiologic and genetic characteristics of 52 CC4821 *N. meningitidis* isolates in Shanghai[abc]

Isolate name	Year	Source	Sg	ST	PFGE type	PorA_VR1	PorA_VR2	'porB	FetA_VR	NHBA-VR	*nhba*	*fHbp*	FHbp variant[d]	*gyrA* allele
NM001	2005	Invasive	C	ST-4821	A	7–2	14	3–48	F3–3	503	124	498	1.80/B	71(R1)
NM002	2006	Contact	C	ST-4821	A	7–2	14	3–48	F3–3	503	124	498	1.80/B	71(R1)
NM010	2006	Invasive	C	ST-9482	A	7–2	14	3–48	F3–3	503	124	512	1.437/B	71(R1)
NM029	2007	Invasive	C	ST-4821	A	7–2	14	3–48	F3–3	503	124	498	1.80/B	71(R1)
NM033	2007	Invasive	C	ST-4821	A	7–2	14	3–48	F3–3	503	124	498	1.80/B	71(R1)
NM045	2008	Invasive	C	ST-4821	A	7–2	14	3–48	F3–3	503	124	498	1.80/B	71(R1)
NM048	2008	Contact	B	ST-9586	A	7–2	14	3–48	F3–3	503	124	22	2.22/A10	71(R1)
NM053	2009	Invasive	C	ST-4821	A	7–2	14	3–48	F3–3	503	124	498	1.80/B	71(R1)
NM061	2011	Contact	C	ST-4821	A	7–2	14	3–48	F3–3	503	124	22	2.22/A10	71(R1)
NM062	2011	Invasive	C	ST-4821	A	7–2	14	3–48	F3–3	503	124	22	2.22/A10	71(R1)
NM119	2011	Invasive	B	ST-9455	A	7–2	14	3–48	F3–3	503	124	498	1.80/B	71(R1)
NM044	2008	Invasive	C	ST-4821	A	7–2	14	3–48	F3–3	503	124	22	2.22/A10	71(R1)
NM055	2010	Invasive	C	ST-4821	A	7–2	14	3–48	F3–3	503	124	498	1.80/B	71(R1)
NM015	2006	Invasive	C	ST-4821	A	7–2	14	3–48	F14–1	503	124	498	1.80/B	71(R1)
NM032	2007	Contact	NG	ST-4821	A	7–2	14	3–48	NP	503	124	22	2.22/A10	71(R1)
NM046	2008	Invasive	C	ST-4821	A	7–2	14	3–606	F3–3	503	124	498	1.80/B	71(R1)
NM042	2007	Invasive	C	ST-9484	A	12–1	16–8	3–48	F3–3	503	124	636	1.546/B	71(R1)

Isolate name	Year	Source	Sg	ST	PFGE type	PorA_VR1	PorA_VR2	'porB	FetA_VR	NHBA-VR	*nhba*	*fHbp*	FHbp variant[d]	*gyrA* allele
NM047	2008	Invasive	C	ST-4821	A	12–1	16–8	3–48	F3–3	503	124	489	1.419/B	71(R1)
NM049	2008	Invasive	C	ST-4821	A	12–1	16–8	3–48	F3–3	503	124	489	1.419/B	71(R1)
NM374	2013	Invasive	C	ST-4821	A	12–1	16–8	3–48	F3–3	503	124	489	1.419/B	71(R1)
NM014	2006	Invasive	C	ST-4821	A	20	23–7	3–48	F3–3	503	124	489	1.419/B	71(R1)
NM039	2007	Invasive	C	ST-4821	A	20	23–7	3–48	F3–3	503	124	489	1.419/B	71(R1)
NM025	2007	Carrier	B	ST-4821	A	20	23–2	3–48	F3–3	503	124	22	2.22/A10	71(R1)
NM023	2007	Carrier	B	ST-9585	B	18	23–6	3–1	F2–9	73	74	16	2.16/A19	12(S1)
NM323	1977	Carrier	B	ST-5798	C	20	23	3–229	F3–9	669	553	16	2.16/A19	12(S1)
NM040	2007	Invasive	B	ST-5798	C	20	13–1	3–370	F5–2	669	553	16	2.16/A19	23(S2)
NM059	2010	Carrier	B	ST-5798	C	20	13–1	3–229	F5–2	669	553	16	2.16/A19	12(S1)
NM050	2009	Contact	B	ST-5798	C	20	13–2	3–229	F5–2	669	553	16	2.16/A19	71(R1)
NM063	2011	Invasive	B	ST-5798	C	20	13–1	3–229	F5–2	669	553	16	2.16/A19	12(S1)
NM200	2011	Carrier	B	ST-5664	D	20	23–9	3–81	F1–112	910	967	16	2.16/A19	12(S1)
NM021	2007	Carrier	B	ST-5664	E	20	23–9	3–81	F1–91	688	965	1207	3.94/A	12(S1)
NM064	2011	Invasive	B	ST-9454	E	20	23–3	3–352	F1–91	688	965	322	1.275/B	113(R13)
NM312	1977	Carrier	C	ST-9591	F	20	23–3	3–35	F4–1	916	973	16	2.16/A19	12(S1)
NM313	1977	Carrier	C	ST-4821	F	20	23–1	3–35	F14–1	669	553	16	2.16/A19	12(S1)
NM325	1977	Carrier	C	ST-5081	F	20	23–7	3–460	F1–7	917	974	16	2.16/A19	12(S1)
NM322	1977	Carrier	C	ST-3436	F	20–3	23–7	3–460	F5–8	697	966	474	2.404/A	12(S1)
NM277	1977	Invasive	C	ST-3436	F	20–3	23–7	3–460	F1–140	697	966	474	2.404/A	12(S1)
NM278	1977	Invasive	C	ST-3436	F	20–3	23–1	3–460	F1–180	697	966	474	2.404/A	12(S1)
NM297	1977	Carrier	B	ST-4821	F	20–1	23–7	3–460	F5–138	669	553	16	2.16/A19	12(S1)
NM193	1972	Carrier	C	ST-3436	F	20–3	23–1	3–607	F1–5	697	966	474	2.404/A	12(S1)
NM213	1976	Carrier	B	ST-4821	F	20	23–1	3–460	F2–30	669	553	16	2.16/A19	12(S1)
NM220	1976	Carrier	B	ST-3436	F	20–3	23–7	3–460	F5–8	697	966	474	2.404/A	12(S1)
NM221	1976	Carrier	B	ST-3436	F	20–3	23–7	3–460	F5–8	697	966	474	2.404/A	12(S1)

Isolate name	Year	Source	Sg	ST	PFGE type	PorA_VR1	PorA_VR2	'porB	FetA_VR	NHBA-VR	*nhba*	*fHbp*	FHbp variant[d]	*gyrA* allele
NM268	1977	Carrier	B	ST-4821	F	20	23–3	3–460	F5–172	915	972	16	2.16/A19	12(S1)
NM319	1977	Carrier	B	ST-3436	F	20–3	23–9	3–609	F5–8	697	966	474	2.404/A	12(S1)
NM205	1973	Carrier	C	ST-4821	F	20	23–2	3–608	F5–135	669	553	16	2.16/A19	4(S2)
NM267	1977	Carrier	B	ST-5798	F	20	23–1	3–48	F5–2	669	553	16	2.16/A19	12(S1)
NM290	1977	Invasive	C	ST-3436	F	20–3	23–3	3–229	F5–8	697	966	474	2.404/A	12(S1)
NM075	2013	Invasive	B	ST-10051	G	20	23–1	3–25	F1–91	9	7	5	1.5/B22	103(R10)
NM038	2007	Contact	B	ST-9502	H	18	25–11	3–229	F3–9	669	553	691	2.585/A	109(R11)
NM282	1977	Carrier	B	ST-3200	H	20	23	3–229	F3–9	669	553	16	2.16/A19	12(S1)
NM057	2005	Invasive	C	ST-4821	I	12–6	2–35	2–142	F1–8	20	808	39	2.18/A	4(S2)

[a] *nadA* is missing in all of the CC4821 strains.

[b] Light pink, lavender blush and light cyan indicated strains belonged to the epidemic clone, non-epidemic clones in phylogenetic group 1 and group 2, respectively, based on the similarity of antigen genes.

[c] Abbreviations: NG, Nongroupable; NP, negative for PCR; Sg, serogroup; ST, sequence type.

[d] FHbp variant: Novartis subvariant/Pfizer subfamily or subvariant. There are three major variants (variant 1, 2 and 3) in Novartis classification for FHbp proteins, named with a major variant number and a subvariant number (e.g., 1.80). Pfizer classifies FHbp into two subfamilies, A and B, with assigned number indicating subvariant (e.g., A10).

Technical Appendix Table 3. List of recombinant genes linked to the divergence of group 1 CC4821 sublineage.

Gene ID	Gene name	Annotation	COG pathway	COG process
NMCC_0117		putative FAD-dependent oxidoreductase	Amino acid transport and metabolism	Metabolism
NMCC_0585	*argH*	argininosuccinate lyase (arginosuccinase; ASAL)	Amino acid transport and metabolism	Metabolism
NMCC_0658	*trpB*	tryptophan synthase β chain	Amino acid transport and metabolism	Metabolism
NMCC_0883	*metX*	homoserine O-acetyltransferase (homoserine O-trans-acetylase; homoserine transacetylase; HTA)	Amino acid transport and metabolism	Metabolism
NMCC_0886	*metF*	5,10-methylenetetrahydrofolate reductase	Amino acid transport and metabolism	Metabolism
NMCC_0887	*metE*	5-methyltetrahydropteroyltriglutamate–homocysteine methyltransferase (methionine synthase, vitamin-B12 independent isoz	Amino acid transport and metabolism	Metabolism
NMCC_1321	*pepN*	aminopeptidase N (α-aminoacylpeptide hydrolase)	Amino acid transport and metabolism	Metabolism
NMCC_1357	*aroD*	3-dehydroquinate dehydratase (3-dehydroquinase; type I DHQase)	Amino acid transport and metabolism	Metabolism
NMCC_1540	*serC*	phosphoserine aminotransferase (PSAT)	Amino acid transport and metabolism	Metabolism
NMCC_1547		putative amino-acid symporter	Amino acid transport and metabolism	Metabolism
NMCC_0140	*ptsIIA*	phosphotransferase system, enzyme IIA (protein IIA)	Carbohydrate transport and metabolism	Metabolism
NMCC_0141	*ptsH*	phosphocarrier protein HPr (phosphotransferase system, histidine-containing protein)	Carbohydrate transport and metabolism	Metabolism
NMCC_0403	*pglD*	pilin glycosylation protein PglD	Carbohydrate transport and metabolism	Metabolism
NMCC_0107	*folD*	FolD bifunctional protein (PPDC) [includes: methylenetetrahydrofolate dehydrogenase and methenyltetrahydrofolate cyclohy	Coenzyme transport and metabolism	Metabolism
NMCC_0114	*thiG*	thiazole biosynthesis protein ThiG	Coenzyme transport and metabolism	Metabolism
NMCC_0115		putative ThiS-like protein	Coenzyme transport and metabolism	Metabolism
NMCC_0116	*thiE*	thiamine-phosphate pyrophosphorylase (TMP pyrophosphorylase; TMP-PPase; thiamine-phosphate synthase)	Coenzyme transport and metabolism	Metabolism
NMCC_0125		putative NAD/FAD binding protein	Coenzyme transport and metabolism	Metabolism
NMCC_1271	*pdxH*	pyridoxamine 5′-phosphate oxidase (PNP/PMP oxidase; PNPOx)	Coenzyme transport and metabolism	Metabolism
NMCC_1453		lactoferrin binding protein B (LbpB)	Coenzyme transport and metabolism	Metabolism
NMCC_1618		truncated putative Tat-translocated NosX-like protein (pseudogene part 2)	Coenzyme transport and metabolism	Metabolism

Gene ID	Gene name	Annotation	COG pathway	COG process
NMCC_2063		putative 5-formyltetrahydrofolate cyclo-ligase (methenyl-THF synthetase)	Coenzyme transport and metabolism	Metabolism
NMCC_0124	*ppc*	phosphoenolpyruvate carboxylase (PEPCase; PEPC)	Energy production and conversion	Metabolism
NMCC_0126	*gpsA*	glycerol-3-phosphate dehydrogenase [NAD(P)+] ((NAD(P)H-dependent glycerol-3-phosphate dehydrogenase)	Energy production and conversion	Metabolism
NMCC_0133	*petA*	ubiquinol-cytochrome c reductase iron-sulfur subunit (Rieske iron-sulfur protein; RISP)	Energy production and conversion	Metabolism
NMCC_0134	*petB*	cytochrome b	Energy production and conversion	Metabolism
NMCC_0135	*petC*	cytochrome c1	Energy production and conversion	Metabolism
NMCC_0378		putative membrane-associated thioredoxin	Energy production and conversion	Metabolism
NMCC_0942		putative oxidoreductase	Energy production and conversion	Metabolism
NMCC_1270		putative iron/sulfur binding oxidoreductase	Energy production and conversion	Metabolism
NMCC_0035		conserved hypothetical integral membrane protein	Inorganic ion transport and metabolism	Metabolism
NMCC_0036		conserved hypothetical lipoprotein	Inorganic ion transport and metabolism	Metabolism
NMCC_0215	*fetB*	enterobactin uptake system binding lipoprotein FetB	Inorganic ion transport and metabolism	Metabolism
NMCC_0841		sulfate/thiosulfate import ATP binding protein CysA (sulfate-transporting ATPase)	Inorganic ion transport and metabolism	Metabolism
NMCC_0842		sulfate transport system permease protein CysW	Inorganic ion transport and metabolism	Metabolism
NMCC_1452	*lbpA*	lactoferrin binding protein A (LbpA)	Inorganic ion transport and metabolism	Metabolism
NMCC_1531	*norB*	nitric oxide reductase	Inorganic ion transport and metabolism	Metabolism
NMCC_0301	*fabD*	malonyl CoA-acyl carrier protein transacylase (MCT)	Lipid transport and metabolism	Metabolism
NMCC_0303	*fabH*	3-oxoacyl-[acyl-carrier-protein] synthase III (β-ketoacyl-ACP synthase III; KAS III)	Lipid transport and metabolism	Metabolism
NMCC_0307	*plsX*	fatty acid/phospholipid synthesis protein PlsX	Lipid transport and metabolism	Metabolism
NMCC_0361	*accB*	biotin carboxyl carrier protein of acetyl-CoA carboxylase (BCCP)	Lipid transport and metabolism	Metabolism
NMCC_0386		putative inner-membrane acyltransferase	Lipid transport and metabolism	Metabolism
NMCC_1457	*fadD*	long-chain-fatty-acid–CoA-ligase (long-chain acyl-CoA synthetase)	Lipid transport and metabolism	Metabolism
NMCC_0118		putative transporter	Nucleotide transport and metabolism	Metabolism

Gene ID	Gene name	Annotation	COG pathway	COG process
NMCC_0299	*guaA*	GMP synthase [glutamine-hydrolyzing] (glutamine amidotransferase; GMP synthetase)	Nucleotide transport and metabolism	Metabolism
NMCC_0383	*fhs*	formate–tetrahydrofolate ligase (formyltetrahydrofolate synthetase; FHS; FTHFS)	Nucleotide transport and metabolism	Metabolism
NMCC_1456	*pyrG*	CTP synthase (UTP–ammonia ligase; CTP synthetase)	Nucleotide transport and metabolism	Metabolism
NMCC_1620	*thyA*	thymidylate synthase (TS; TSase)	Nucleotide transport and metabolism	Metabolism
NMCC_2067	*pyrH*	uridylate kinase (UK; uridine monophosphate kinase; UMP kinase)	Nucleotide transport and metabolism	Metabolism
NMCC_0882		putative methionine biosynthesis protein MetW	Secondary metabolites biosynthesis, transport and catabolism	Metabolism
NMCC_1874		putative methyltransferase	Secondary metabolites biosynthesis, transport and catabolism	Metabolism
NMCC_0136		putative RmuC-like protein	Replication, recombination and repair	Information storage and processing
NMCC_0398	*dnaE*	DNA polymerase III α subunit	Replication, recombination and repair	Information storage and processing
NMCC_0619	*ligA*	DNA ligase (polydeoxyribonucleotide synthase [NAD+])	Replication, recombination and repair	Information storage and processing
NMCC_0657		putative DNA glycosylase	Replication, recombination and repair	Information storage and processing
NMCC_0688	*ihfA*	integration host factor α-subunit (IHF-α)	Replication, recombination and repair	Information storage and processing
NMCC_0735		putative deoxyribonuclease	Replication, recombination and repair	Information storage and processing
NMCC_1114	*recD*	exodeoxyribonuclease V α chain	Replication, recombination and repair	Information storage and processing
NMCC_1322		putative polynucleotidyl transferase	Replication, recombination and repair	Information storage and processing

Gene ID	Gene name	Annotation	COG pathway	COG process
NMCC_1325	*ruvC*	crossover junction endodeoxyribonuclease RuvC (Holliday junction nuclease RuvC; Holliday junction resolvase RuvC)	Replication, recombination and repair	Information storage and processing
NMCC_1871	*recQ*	ATP-dependent DNA helicase RecQ	Replication, recombination and repair	Information storage and processing
NMCC_1326		putative Fis-like DNA binding protein	Transcription	Information storage and processing
NMCC_0003	*gltX*	glutamyl-tRNA synthetase (Glutamate–tRNA ligase; GluRS)	Translation, ribosomal structure and biogenesis	Information storage and processing
NMCC_0031	*metG*	methionyl-tRNA synthetase (methionine–tRNA ligase; MetRS)	Translation, ribosomal structure and biogenesis	Information storage and processing
NMCC_0363	*queA*	S-adenosylmethionine:tRNA ribosyltransferase-isomerase (queuosine biosynthesis protein QueA)	Translation, ribosomal structure and biogenesis	Information storage and processing
NMCC_0384		putative GTP-dependent nucleic acid binding protein EngD	Translation, ribosomal structure and biogenesis	Information storage and processing
NMCC_0387	*tyrS*	tyrosyl-tRNA synthetase (tyrosine–tRNA ligase; TyrRS)	Translation, ribosomal structure and biogenesis	Information storage and processing
NMCC_0656	*ksgA*	dimethyladenosine transferase (S-adenosylmethionine-6-N', N'-adenosyl(rRNA) dimethyltransferase; 16S rRNA dimethylase; h	Translation, ribosomal structure and biogenesis	Information storage and processing
NMCC_0687	*pheT*	phenylalanyl-tRNA synthetase β chain (phenylalanine–tRNA ligase β chain; PheRS)	Translation, ribosomal structure and biogenesis	Information storage and processing
NMCC_0880	*efp*	elongation factor P (EF-P)	Translation, ribosomal structure and biogenesis	Information storage and processing
NMCC_0884		50S ribosomal protein L36	Translation, ribosomal structure and biogenesis	Information storage and processing
NMCC_0885	*rpmE*	50S ribosomal protein L31 type B	Translation, ribosomal structure and biogenesis	Information storage and processing

Gene ID	Gene name	Annotation	COG pathway	COG process
NMCC_1267	*gatA*	aspartyl-tRNA(Asn) amidotransferase subunit A (Asp-ADT subunit A)	Translation, ribosomal structure and biogenesis	Information storage and processing
NMCC_1269	*gatB*	aspartyl-tRNA(Asn) amidotransferase subunit B (Asp-ADT subunit B)	Translation, ribosomal structure and biogenesis	Information storage and processing
NMCC_1327	*dusB*	tRNA-dihydrouridine synthase B	Translation, ribosomal structure and biogenesis	Information storage and processing
NMCC_1458	*trmU*	tRNA (5-methylaminomethyl-2-thiouridylate)-methyltransferase	Translation, ribosomal structure and biogenesis	Information storage and processing
NMCC_2065	*rpsB*	30S ribosomal protein S2	Translation, ribosomal structure and biogenesis	Information storage and processing
NMCC_2066	*tsf*	elongation factor Ts (EF-Ts)	Translation, ribosomal structure and biogenesis	Information storage and processing
NMCC_0376		putative ATP binding protein	Cell cycle control, cell division, chromosome partitioning	Cellular processes and signaling
NMCC_0659		IgA-specific serine endopeptidase (IgA protease)	Cell cycle control, cell division, chromosome partitioning	Cellular processes and signaling
NMCC_0985	*ftsK*	DNA translocase FtsK2	Cell cycle control, cell division, chromosome partitioning	Cellular processes and signaling
NMCC_0417	*pilP*	type IV pilus biogenesis lipoprotein PilP	Cell motility	Cellular processes and signaling
NMCC_0418	*pilO*	type IV pilus biogenesis protein PilO	Cell motility	Cellular processes and signaling
NMCC_0419	*pilN*	type IV pilus biogenesis protein PilN	Cell motility	Cellular processes and signaling
NMCC_0032	*glmS*	glucosamine–fructose-6-phosphate aminotransferase [isomerizing] (hexosephosphate aminotransferase; D-fructose-6- phosph	Cell wall/membrane/envelope biogenesis	Cellular processes and signaling

Gene ID	Gene name	Annotation	COG pathway	COG process
NMCC_0034	*mltA*	membrane-bound lytic murein transglycosylase A (murein hydrolase A; GNA33)	Cell wall/membrane/envelope biogenesis	Cellular processes and signaling
NMCC_1324		putative lipid A biosynthesis (KDO)2-(lauroyl)-lipid IVA acyltransferase	Cell wall/membrane/envelope biogenesis	Cellular processes and signaling
NMCC_1616	*rfaK*	α 1,2 N-acetylglucosamine transferase	Cell wall/membrane/envelope biogenesis	Cellular processes and signaling
NMCC_1685	*murI*	glutamate racemase	Cell wall/membrane/envelope biogenesis	Cellular processes and signaling
NMCC_0300	*msbA*	lipid A export ATP binding/permease protein MsbA	Defense mechanisms	Cellular processes and signaling
NMCC_1115	*lolD*	lipoprotein-releasing system ATP binding protein LolD	Defense mechanisms	Cellular processes and signaling
NMCC_0448	*tpsB*	TpsA activation/secretion protein TpsB	Intracellular trafficking, secretion, and vesicular transport	Cellular processes and signaling
NMCC_0350		putative peptidase	Posttranslational modification, protein turnover, chaperones	Cellular processes and signaling
NMCC_0843	*cysT*	sulfate transport system permease protein CysT	Posttranslational modification, protein turnover, chaperones	Cellular processes and signaling
NEIMB_0895		hypothetical protein		
NMCC_0033		hypothetical protein		
NMCC_0304		hypothetical protein		
NMCC_0305				
NMCC_0306		hypothetical protein		
NMCC_0442		Cupin superfamily protein		
NMCC_0686		hypothetical protein		
NMCC_0689		hypothetical protein		
NMCC_0878		hypothetical protein		

Gene ID	Gene name	Annotation	COG pathway	COG process
NMCC_0881		hypothetical protein		
NMCC_1539		hypothetical protein		
NMCC_1619		hypothetical protein		
NMCC_1872		hypothetical protein		
NMCC_1873		hypothetical integral membrane protein		
NMCC_2064		hypothetical protein		
NMCC_0302		conserved hypothetical integral membrane protein	Function unknown	Poorly characterized
NMCC_0879		conserved hypothetical protein	Function unknown	Poorly characterized
NMCC_1268		conserved hypothetical protein	Function unknown	Poorly characterized
NMCC_1541		conserved hypothetical protein	Function unknown	Poorly characterized
NMCC_1548		conserved hypothetical protein	Function unknown	Poorly characterized
NMCC_0369		putative phosphoribosyltransferase	General function prediction only	Poorly characterized
NMCC_0690		putative FxsA-like protein	General function prediction only	Poorly characterized
NMCC_1538		putative sulfatase	General function prediction only	Poorly characterized
NMCC_1617		putative sodium-dependent transporter	General function prediction only	Poorly characterized
NMCC_1875	*bioH*	carboxylesterase BioH (biotin synthesis protein BioH)	General function prediction only	Poorly characterized
NMCC_1876		putative GntX-like protein	General function prediction only	Poorly characterized

Technical Appendix Table 4. List of genes found in majority of group 1 CC4821 strains but not in group 2

Gene ID	Gene name	Annotation
GNM001_00395		Cupin superfamily protein
GNM001_01225		Hypothetical protein
NEIMB_0683	*nhaP*	K(+)/H(+) antiporter NhaP
NMCC_0368		Hypothetical protein
NMCC_0369		Phosphoribosyl transferase domain protein
NMCC_0638		Hypothetical protein
NMCC_0790		Putative type I restriction enzymeP M protein
NMCC_1121	*mgsR*	Regulatory protein MgsR
NMCC_1206		Hypothetical protein
NMCC_1453	*lbpB*	Lactoferrin binding protein B (LbpB)
NMCC_2037	*ymdB*	O-acetyl-ADP-ribose deacetylase

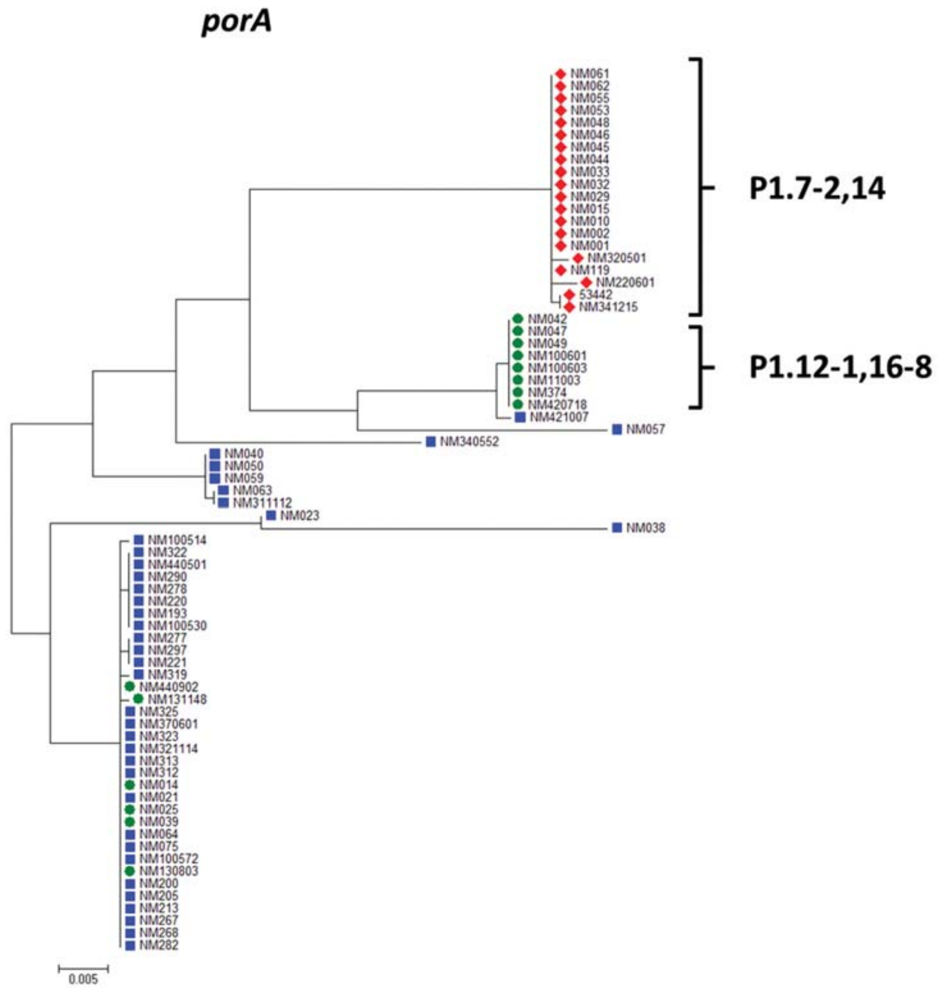

Technical Appendix Figure 1. Phylogenetic analysis of antigen gene sequence variation within the *Neisseria meningitidis* clonal complex 4821 lineage, based on nucleotide sequences of the *porA* gene. Phylogenetic trees of aligned antigen gene sequences for clonal complex 4821 isolates show extensive sequence diversity across all 5 antigen genes, most prominently among group 2 isolates (blue squares) compared with group 1 isolates (red diamonds and green dots). Group 1 isolates have unique *nada* and *fetA* alleles, whereas the epidemic clone (red diamonds) has acquired a genetically distinct *porA* genotype P1.7–2, 14 compared with remaining group 1 isolates (green dots). Scale bars represent total substitutions per site.

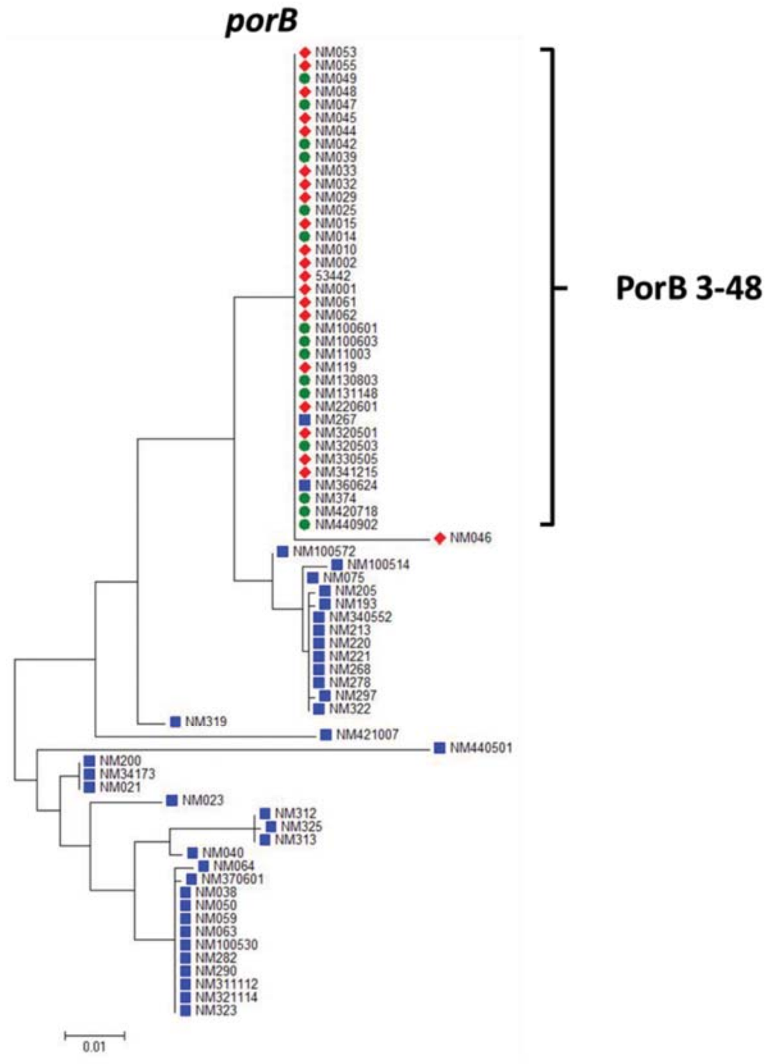

Technical Appendix Figure 2. Phylogenetic analysis of antigen gene sequence variation within the *Neisseria meningitidis* clonal complex 4821 lineage, based on nucleotide sequences of the *porB* gene. Phylogenetic trees of aligned antigen gene sequences for clonal complex 4821 isolates show extensive sequence diversity across all 5 antigen genes, most prominently among group 2 isolates (blue squares) compared with group 1 isolates (red diamonds and green dots). Group 1 isolates have unique *nada* and *fetA* alleles, whereas the epidemic clone (red diamonds) has acquired a genetically distinct *porA* genotype P1.7–2, 14 compared with remaining group 1 isolates (green dots). Scale bars represent total substitutions per site.

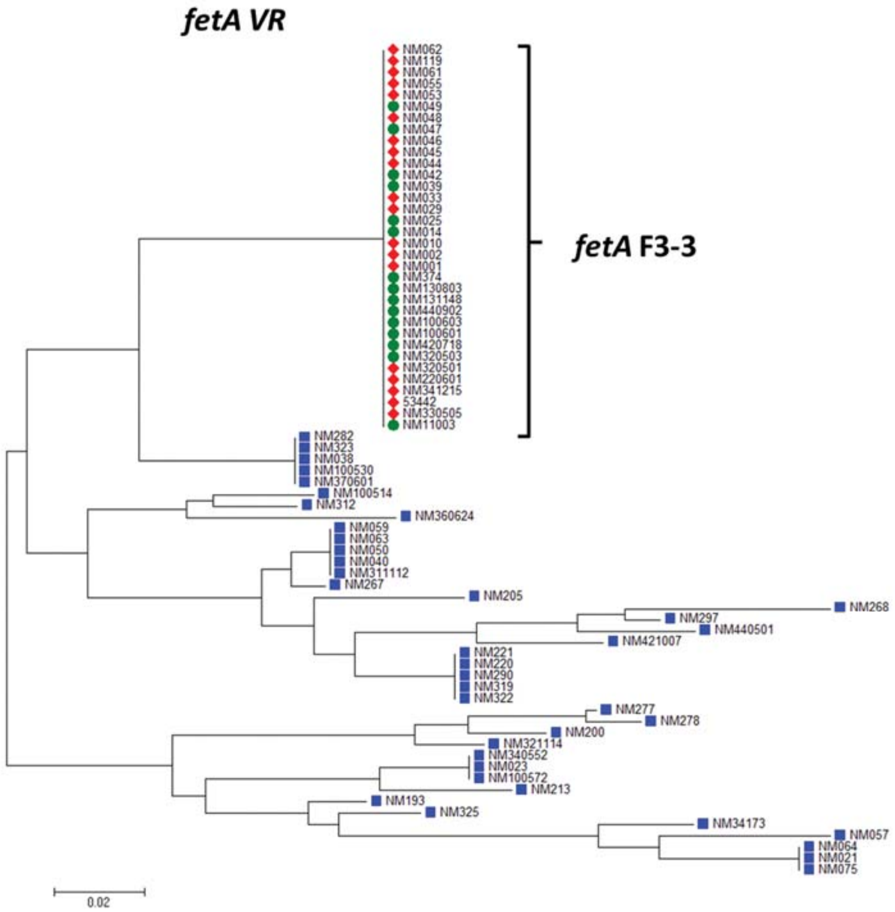

Technical Appendix Figure 3. Phylogenetic analysis of antigen gene sequence variation within the *Neisseria meningitidis* clonal complex 4821 lineage, based on nucleotide sequences of the *fetA* gene. Phylogenetic trees of aligned antigen gene sequences for clonal complex 4821 isolates show extensive sequence diversity across all 5 antigen genes, most prominently among group 2 isolates (blue squares) compared with group 1 isolates (red diamonds and green dots). Group 1 isolates have unique *nada* and *fetA* alleles, whereas the epidemic clone (red diamonds) has acquired a genetically distinct *porA* genotype P1.7–2, 14 compared with remaining group 1 isolates (green dots). Scale bars represent total substitutions per site.

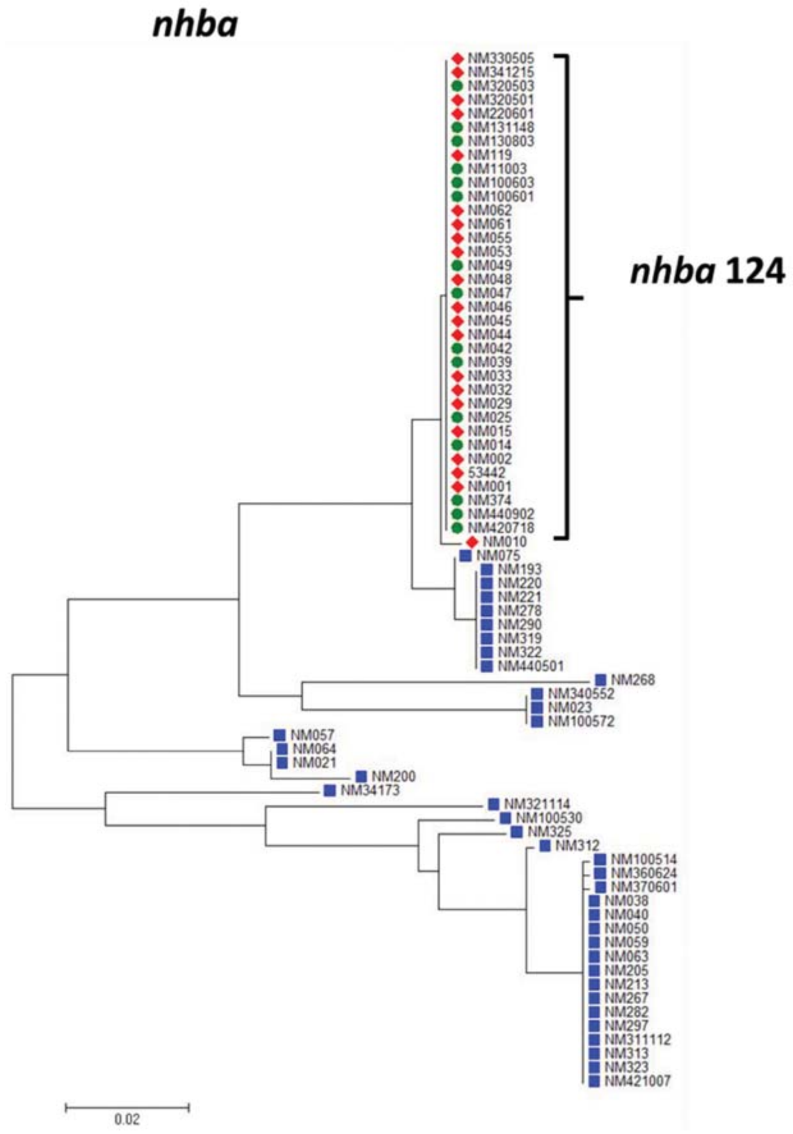

Technical Appendix Figure 4. Phylogenetic analysis of antigen gene sequence variation within the *Neisseria meningitidis* clonal complex 4821 lineage, based on nucleotide sequences of the *nhba* gene. Phylogenetic trees of aligned antigen gene sequences for clonal complex 4821 isolates show extensive sequence diversity across all 5 antigen genes, most prominently among group 2 isolates (blue squares) compared with group 1 isolates (red diamonds and green dots). Group 1 isolates have unique *nada* and *fetA* alleles, whereas the epidemic clone (red diamonds) has acquired a genetically distinct *porA* genotype P1.7–2, 14 compared with remaining group 1 isolates (green dots). Scale bars represent total substitutions per site.

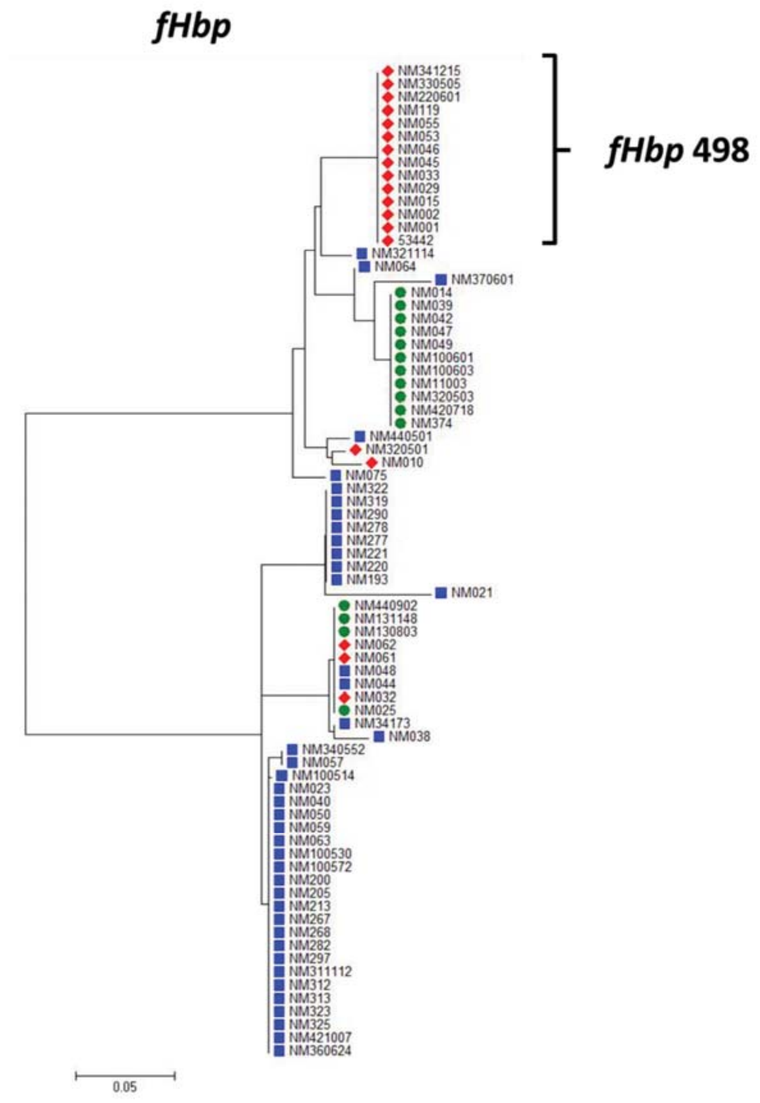

Technical Appendix Figure 5. Phylogenetic analysis of antigen gene sequence variation within the *Neisseria meningitidis* clonal complex 4821 lineage, based on nucleotide sequences of the *fHbp* gene. Phylogenetic trees of aligned antigen gene sequences for clonal complex 4821 isolates show extensive sequence diversity across all 5 antigen genes, most prominently among group 2 isolates (blue squares) compared with group 1 isolates (red diamonds and green dots). Group 1 isolates have unique *nada* and *fetA* alleles, whereas the epidemic clone (red diamonds) has acquired a genetically distinct *porA* genotype P1.7–2, 14 compared with remaining group 1 isolates (green dots). Scale bars represent total substitutions per site.
